# On the Regulatory Approval Pathway of Biosimilar Products

**DOI:** 10.3390/ph5040353

**Published:** 2012-03-30

**Authors:** Jun Wang, Shein-Chung Chow

**Affiliations:** 1 Duke University School of Medicine, DUMC 3813, Durham, NC 27710, USA; 2 Duke University School of Medicine, Duke Box 2721, Durham, NC 27710, USA; Email: sheinchung.chow@duke.edu (J.W.)

**Keywords:** biosimilars, biosimilarity, interchangeability, WHO, EMA, FDA

## Abstract

Biosimilars (or follow-on biologics) are a new class of medicine which enters the market subsequent to a previously approved version. They have demonstrated similarity to innovator biologic products in terms of quality, safety, and efficacy. The EMA has taken the lead in the regulatory approval framework for biosimilar products, and WHO has published guidelines on the evaluation of biosimilars in order to facilitate the global harmonization. Based on EMA and WHO guidelines, many other countries such as Canada, Japan and Korea have also issued their own guidance for evaluating follow-on biologics. The US FDA was authorized to approve follow-on biologics by the BPCI Act passed by the US Congress on March 23, 2010, and has just issued a draft guidance in early 2012. The basic concepts and main principles of approving biosimilars are similar among various nations, notwithstanding some differences in regard to the scope, the choice of reference product, and the data requirement. This article reviews the regulatory approval pathway of biosimilar products in different regions.

## 1. Introduction

When an innovator (brand-name) drug product is going off patent, pharmaceutical companies may file an abbreviated new drug application (ANDA) for approval of the generic copies of the innovator drug product. In 1984, the US Food and Drug Administration (FDA) was authorized to approve generic drug products under the Drug Price Competition and Patent Term Restoration Act, (also known as the ‘Hatch-Waxman Act’). According to FDA’s definition, the generic drug products should be comparable to the reference drug product in dosage form, strength, route of administration, quality, performance characteristics, and intended use. Most regulatory agencies have established one size-fits-all criterion of the approval of generic (small molecule) drug products, which requires that evidence of average bioavailability be provided through the conduct of bioequivalence studies. The assessment of bioequivalence as a surrogate for the evaluation of drug safety and efficacy is based on the so-called Fundamental Bioequivalence Assumption that if two drug products are shown to be bioequivalent in average bioavailability, it is assumed that they will reach the same therapeutic effect and hence can be used interchangeably [1]. As indicated in the FDA guidance for the assessment of bioequivalence, bioequivalence usually can be assessed under a standard 2 × 2 crossover design through log-transformed data using the bioequivalence limit of (80%, 125%). In such cases, two drug products can be claimed bioequivalent in average bioavailability if the 90% confidence interval of the ratio of means of the primary pharmacokinetic response such as the AUC (area under the blood or plasma concentration-time curve) or C_max_ (maximum concentration) is totally within (80%, 125%) [2]. In sum, the regulatory requirements and statistical methods for the assessment of bioequivalence for generic (small molecule) drugs are well established.

With fast and advanced development of modern biological technology especially recombinant DNA technology, biologic drug products have played more and more important roles in treating many life-threatening and chronic diseases. As a result, biologic drugs have comprised a growing segment in the pharmaceutical industry. For instance, global sales of biologics were $93 billion in 2009, and these sales are expected to continue to grow at least twice as fast as those of small molecules [3]. However, the high unit cost of biologics has resulted in patients’ concerns about continued access to potentially effective therapies. Recently, the expiration of patents for a number of blockbuster biologics has ushered in an era of the subsequent production of biosimilar products, which might contribute to increased access to these products at an affordable price. However, unlike small molecular drugs with clearly and well-defined composition and structure, follow-on biologic products that are made in or isolated from living systems have much more complex ingredients. Therefore, there is general consensus that the standard methodology for the assessment of bioequivalence is not appropriate for the assessment of biosimilarity of follow-on biologics, which highlights the need of more complex and specified regulation and approval tracks.

The purpose of this article is to review regulatory requirements for the approval pathway of follow-on biologics worldwide including WHO and various regions, such as European Union, US, Canada, and Asian Pacific Region (e.g., Japan and Korea). Comparison of these regulatory requirements, and some recommendations regarding global harmonization will be made. In the next section, definitions and interpretations of biosimilar products from different regions are given. Regulatory requirements from different regions are briefly summarized in Section 3. Recommendations on global harmonization of regulatory approval pathways are given in Section 4. Section 5 provides some concluding remarks. 

## 2. Definitions and Interpretations of Biosimilar Products

The similar biologic drug products are usually referred to as similar biotherapeutic product (SBPs) by WHO, biosimilars by European Medicines Agency (EMA) of the European Union (EU), follow-on biologics (FOBs) by the US FDA, and subsequent-entry biologics (SEBs) by Health Canada. In some cases, the term “biosimilar” has been used in an inappropriate way, and therefore it is important to review differences in definitions of biosimilar products in different regions (see Table 1).

WHO defines SBP as a biotherapeutic product, which is similar in terms of quality, safety and efficacy to an already licensed reference biotherapeutic product [4]. Health Canada defines biosimilar to be a biologic drug that enters the market subsequent to a version previously authorized in Canada, and with demonstrated similarity to a reference biologic drug [5]. As indicated in the Biologics Price Competition and Innovation (BPCI) Act passed by the US Congress on March 23, 2010, a biosimilar product is defined as a product that is highly similar to the reference product, notwithstanding minor differences in clinically inactive components, and for which there are no clinically meaningful differences in terms of safety, purity, and potency from the reference product. EMA did not provide the definition of biosimilars in the original guidelines. However, a recently published concept paper on the revision of the guidelines on similar biological medicinal product indicated that it might be prudent to discuss if a definition of “biosimilar”, in extension of what is in the legislation and relevant CHMP (Committee for Medicinal Products for Human Use) guidance, is necessary [6]. 

**Table 1:** Definitions of biosimilar products.

Term	By	Definition
**SBP**	WHO	A biotherapeutic product similar to an already licensed reference biotherapeutic product in terms of quality, safety and efficacy
**FOB**	US FDA	A product highly similar to the reference product without clinically meaningful differences in safety, purity and potency
**SEB**	Canada	A biologic drug that enters the market subsequent to a version previously authorized in Canada with demonstrated similarity to a reference biologic drug
**Biosimilar**	Korea	Biological products which demonstrated its equivalence to an already approved reference product with regard to quality, safety, and efficacy

Based on these different definitions, we would interpret that there are three determinants in the definition of the biosimilar product: (1) it should be a biologic product; (2) the reference product should be an already licensed biologic product; (3) the demonstration of high similarity in safety, quality, and efficacy is necessary. Besides, it is well recognized that the similarity should be demonstrated using a set of comprehensive comparability exercises at the quality, non-clinical and clinical level. Products not authorized by this comparability regulatory pathway cannot be called biosimilars.

## 3. Regulatory Requirements

As indicated by Chow *et al.* [7], standard methods for the assessment of bioequivalence for generic drug products with identical active ingredients are not appropriate for the assessment of biosimilarity due to the fundamental differences between small molecule drug products and biologic products. For the assessment of follow-on biologics, regulatory requirements from different regions such as European Union (EU), US, and Asian Pacific Region are similar and yet slightly different. In what follows, these regulatory requirements will be described. 

### 3.1. World Health Organization (WHO)

As an increasingly wide range of SBPs are under development or are already licensed in many countries, WHO formally recognized the need for the guidance for their evaluation and overall regulation in 2007. “Guidelines on Evaluation of Similar Biotherapeutic Products (SBPs)” was developed and adopted by the 60^th^ meeting of the WHO Expert Committee on Biological Standardization in 2009. The intention of the guidelines is to provide globally acceptable principles for licensing biotherapeutic products that are claimed to be similar to the reference products that have been licensed based on a full licensing dossier. [4] The scope of the guidelines includes well-established and well-characterized biotherapeutic products that have been marketed for a suitable period of time with a proven quality, efficacy and safety, such as recombinant DNA-derived therapeutic proteins. 

#### 3.1.1. Key Principles and Basic Concept

Key principles and basic concept for licensing SBPs have been explained in WHO’s guidelines. One of the most important principles of developing SBP is the stepwise approach starting with characterization of quality attributes of the product and followed by non-clinical and clinical evaluations. Manufacturers should submit a full quality dossier that includes a complete characterization of the product, the demonstration of consistent and robust manufacture of their product, and the comparability exercise between the SBP and the reference biotherapeutic product (RBP) in the quality part, which together serve as the basis for the possible reduction in data requirement in the non-clinical and clinical development. This principle indicates that the data reduction is only possible for the non-clinical and clinical parts of the development program, and significant differences between the SBP and the chosen RBP detected during the comparability exercise would result in requirement for more extensive non-clinical and clinical data. In addition, the amount of non-clinical and clinical data considered necessary is also dependent on the class of products, which calls for a case by case approach for different classes of products. 

#### 3.1.2. Reference Biotherapeutic Product

The choice of the reference biotherapeutic product is another important issue covered in the WHO’s guidelines. Traditionally, National Regulatory Authorities (NRA) have required the use of a nationally licensed reference product for licensing of generic medicines, but this may not be feasible for countries lacking nationally-licensed RBPs. Thus additional criteria to guide the acceptability of using a RBP licensed in other jurisdiction may be needed. Considering the choice of the RBP, WHO requires that it should have been marketed for a suitable duration and have a volume of marketed use, and should be licensed based on a full quality, safety, and efficacy data. Besides, same RBP should be used throughout the development of SBP, and the drug substance, dosage form and route of administration of SBP should be the same as that of RBP. 

#### 3.1.3. Quality

As mentioned in a former section, the comprehensive comparison showing similarity of quality between SBP and RBP is a prerequisite for applying the clinical safety and efficacy profile of RBP to SBP, thus a full quality dossier for both drug substance and drug product is always required. To evaluate comparability, WHO recommends the manufacturer to conduct a comprehensive physicochemical and biological characterization of the SBP in head-to-head comparisons with RBP. The following aspects of product quality and heterogeneity should be assessed.

##### Manufacturing Process

The manufacturing process should meet the same standards as required by NRA for originator products, and implement Good Manufacturing Practices, modern quality control and assurance procedures, in-process controls, and process validation. The SBP manufacturer should assemble all available knowledge of the RBP with regard to the type of host cell, formulation and container closure system, and submit a complete description and data package delineating the whole manufacturing process including obtaining and expression of target genes, the optimization and fermentation of gene engineering cells, the clarification and purification of the products, the formulation and testing, aseptic filling and packaging. 

##### Characterization

Thorough characterization and comparability exercise are required, and details should be provided on primary and higher-order structure, post-translational modifications, biological activity, process- and product-related impurities, the relevant immunochemical properties, and results from accelerated degradation studies and studies under various stress conditions. 

#### 3.1.4. Non-Clinical and Clinical Studies

After demonstrating the similarity of SBP and RBP in quality, the proving of safety and efficacy of a SBP usually requires further non-clinical and clinical data. Non-clinical evaluations should be undertaken both *in vitro* (e.g., receptor-binding studies, cell-proliferation, cytotoxicity assays) and *in vivo* (e.g., biological/ pharmacodynamic activity, repeat dose toxicity study, toxicokinetic measurements, anti-product antibody titers, cross reactivity with homologous endogenous proteins, product neutralizing capacity). 

In terms of the clinical evaluation, the comparability exercise should begin with pharmacokinetic (PK) and pharmacodynamic (PD) studies followed by the pivotal clinical trials. PK studies should be designed to enable detection of potential differences between SBP and RBP. Single-dose, cross-over PK studies in homogenous population are recommended by WHO. The manufacturer should justify the choice of single-dose studies, steady-state studies, or repeated determination of PK parameters, and the study population. Due to the lack of established acceptance criteria for the demonstration of similar PK between SBP and RBP, the traditional 80-125% equivalence range is often used. Besides, PD studies and confirmatory PK/PD studies may be appropriate if there are clinically relevant PD markers. In addition, similar efficacy of SBP and RBP has to be demonstrated in randomized and well-controlled clinical trials, which should preferably be double-blind or at least observer-blind. In principle, equivalence designs (requiring lower and upper comparability margins) are clearly preferred for the comparison of efficacy and safety of SBP with RBP. Non-inferiority designs (requiring only one margin) may be considered if appropriately justified. WHO also suggest the pre-licensing safety data and the immunogenicity data should be obtained from the comparative efficacy trials. 

In addition to the non-clinical and clinical data, applicants also need to present an ongoing risk management and pharmacovigilance plan, since data from pre-authorized clinical studies are usually too limited to identify all potential side effects of the SBP. The safety specification should describe important identified or potential safety issues for the RBP, and any that are specific for the SBP. 

In sum, the WHO guidelines on evaluating similar biotherapeutic products represent an important step forward in the global harmonization for the evaluation and regulation of biosimilar products, and provide clear guidance for both regulatory bodies and the pharmaceutical industry. 

### 3.2. European Union (EU)

The European Union (EU) has pioneered in the development of a regulatory system for biosimilar products. The European Medicines Agency (EMA) began formal consideration of scientific issues presented by biosimilar products at least as early as January 2001, when an *ad hoc* working group discussed the comparability of medicinal products containing biotechnology-derived proteins as active substances [8]. In 2003, the European Commission amended the provisions of the EU secondary legislation governing requirements for marketing authorization applications for medicinal products and established a new category of applications for “similar biological medicinal products” [9]. In 2005, the EMA issued a general guideline on similar biological medicinal products, in order to introduce the concept of similar biological medicinal products, to outline the basic principles to be applied, and to provide applicants with a ‘user guide’, showing where to find relevant scientific information [10]. Since then, 13 biosimilar products have been approved by EMA under the pathway. Two of them are somatropins, five are epoetins, and six are filgrastims. One of the rejected biosimilars is Alpheon (interferon alfa-2a). It was developed by BioPartners GmbH, and designed to become a biosimilar of the reference product Roferon-A for the treatment of adult patients with chronic hepatitis C. The EMA refused the marketing authorization for Alpheon due to the difference identified between Alpheon and the reference product, such as impurities, stability, and side effects. 

#### 3.2.1. Key Principles and Basic Concept

Unlike WHO’s guideline which seems to focus more on recombinant DNA-derived therapeutic proteins, EMA’s guidelines clearly indicate that the concept of a ‘similar biological medicinal product’ is applicable to a broad spectrum of products ranging from biotechnology-derived therapeutic proteins to vaccines, blood-derived products, monoclonal antibodies, gene and cell-therapy, etc. However, comparability exercises to demonstrate similarity are more likely to be applied to highly purified products, which can be thoroughly characterized, such as biotechnology-derived medicinal products. Considering the amount of data submitted, EMA also requires a full quality dossier, while the comparability exercise at the quality level may allow a reduction of the non-clinical and clinical data requirement compared to a full dossier. In 2011, a concept paper on the revision of the guideline on similar biological medicinal products was published by EMA [11], which emphasized another main concept that clinical benefit has already been established by the reference medicinal product, and that the aim of a biosimilar development program is to establish similarity to the reference product, not clinical benefit. Besides, a clear definition of ‘biosimilar’, the feasibility to follow the generic legal basis for some biological products, and the refinement based on the experience gained are recommended. It is anticipated that the draft revised guideline will be released for consultation in the first semester of 2012. 

#### 3.2.2. Reference Biotherapeutic Product

Similarly to the WHO, EMA requires that the active substance, the pharmaceutical form, strength, route of administration of the biosimilar should be the same as that of the reference product. The same chosen reference medicinal product should be used throughout the comparability program for quality, safety, and efficacy studies during the development of the biosimilar product. One of the major differences between WHO and EMA in terms of the choice of reference product is that EMA requires the chosen reference medicinal product to be a medicinal product authorized in the EU. Data generated from comparability studies with medicinal products authorized outside the EU may only provide supportive information. 

#### 3.2.3. Quality

In 2006, the “Guideline on Similar Biological Medicinal Products Containing Biotechnology-derived Proteins as Active Substance: Quality Issues” was adopted by the CHMP [12], which addressed the requirements regarding manufacturing processes, the comparability exercises for quality, analytical methods, physicochemical characterization, biological activity, purity and specifications of the similar biological medicinal product. In 2011, EMA issued a concept paper on the revision of this guideline [13]. This concept paper proposes that the guideline published in 2006 needs refinements taking into account the evolution of quality profile during the product lifecycle, since in the context of a biotherapeutic product claiming or claimed to be similar to another one already marketed, the conclusion of a comparability exercise performed with a reference product at a given time may not hold true from the initial development of the biosimilar, through marketing authorization, until the product’s discontinuation. 

#### 3.2.4. Non-Clinical and Clinical Evaluation

The “Guideline on Similar Biological Medicinal Products Containing Biotechnology-derived Proteins as Active Substance: Non-clinical and Clinical Issues”, published in 2006, lays down the non-clinical and clinical requirements for a biological medicinal product claiming to be similar to another one already marketed [14]. The non-clinical section of the guideline addresses the pharmaco-toxicological assessment, and the clinical section addresses the requirements for pharmacokinetic, pharmacodynamic and efficacy studies. Clinical safety studies as well as the risk management plan with special emphasis on studying the immunogenicity of the biosimilar products are also required. In 2011, EMA published a concept paper on the revision of this guideline [15], which indicates several issues that need discussion for a potential revision. Firstly, EMA emphasizes the need to follow the 3R principles (replacement, reduction and refinement) with regard to the use of animal experiments. Secondly, a revised version of the guideline will consider a risk-based approach for the design of an appropriate non-clinical study program. Thirdly, the guideline should be clearer considering the need and acceptance of pharmacodynamics markers, and what measures should be taken in case relevant markers are not available. The draft guideline will probably be released for consultation in the first semester of 2012. 

#### 3.2.5. Product Class-Specific Guidelines

The principles of biosimilar drug development discussed in the former sections apply in general to all biological drug products. However, there are no standard data sets that can be applied to the approval of all classes of biosimilars. Each class of biologic varies in its benefit/risk profile, the nature and frequency of adverse events, the breadth of clinical indications, and whether surrogate markers for efficacy are available and validated. Accordingly, the EMA has developed product class-specific guidelines that define the nature of comparative studies. So far, guidance for the development of biosimilar products has been developed for six different product classes, including erythropoietins, insulins, growth hormones, alfa interferons, granulocyte-colony stimulating factors and low-molecular weight heparins (LMWH), with three more (beta interferons, follicle stimulation hormone, monoclonal antibodies) currently being drafted [16,17,18,19,20,21,22,23,24]. 

In summary, the EU has taken a thoughtful and evidence-based approach, and has established a well-documented legal and regulatory pathway for the approval of biosimilar products distinct from the generic pathway. In order to grant a biosimilar product, the EMA requires comprehensive and justified comparability studies between the biosimilar and the reference products in the quality, non-clinical, and clinical level, which are explained in detail in the EMA guidelines. The approval pathway of biosimilar products in the EU is based on case-by-case reviews, owing to the complexity and diversity of the biologic products. Therefore, besides the three general guidelines, EMA also developed additional product class-specific guidelines on non-clinical and clinical studies. This approval pathway is now held up as one of the gold standards for authorizing biosimilar products. 

### 3.3. North America (US & Canada)

#### 3.3.1. US (FDA)

For the approval of follow-on biologics in the United States, current regulations depends on whether the biologic product is approved under the United States Food, Drug, and Cosmetic Act (US FD&C) or it is licensed under the United States Public Health Service Act (US PHS). For those biologic drugs marketed under the PHS Act, the BPCI Act passed by the US Congress on March 23, 2010 amends the PHS Act to establish an abbreviated approval pathway for biological products that are highly similar or interchangeable with an FDA-authorized biologic drug, and gives the FDA the authority to approve follow-on biologics under new section 351(k) of the PHS Act. Some early biologic drugs, such as somatropin and insulin were approved under the FD&C Act. In this case, biosimilar versions can receive approval for New Drug Applications (NDAs) under section 505 (b)(2) of the FD&C Act. 

Following the passage of the BPCI, in order to obtain input on specific issues and challenges associated with the implementation of the BPCI Act from a broad group of stakeholders, the US FDA conducted a two-day public hearing on Approval Pathway for Biosimilar and Interchangeable Biological Products held on November 2-3, 2010 at the FDA in Silver Spring, Maryland. The scientific issues covered in this public hearing included, but not limited to, criteria and design for biosimilarity and interchangeability, comparability between manufacturing processes, patient safety and pharmacovigilance, exclusivity and user fees. 

In practice, there is a strong industrial interest and desire for the regulatory agencies to develop review standards and an approval process for biosimilars rather than an *ad hoc* case-by-case review of individual biosimilar applications. For this purpose, the FDA has established three committees to ensure consistency in the FDA’s regulatory approach of follow-on biologics. The three committees are the Center for Drug Evaluation and Research (CDER)/Center for Biologics Evaluation and Research (CBER) Biosimilar Implementation Committee (BIC), the CDER Biosimilar Review Committee (BRC), and the CBER Biosimilar Review Committee. The CDER/CBER BRC will focus on the cross-center policy issues related to the implementation of the BPCI Act. The CDER BRC and CBER BRC are responsible for considering requests of applicants for advice about proposed development programs for biosimilar products, reviewing Biologic License Applications (BLAs) that are submitted under section 351(k) of the PHS Act, and managing related issues. Thus, the review process steps of CDER BRC and CBER BRC include: (1) applicant submits request for advice, (2) internal review team meeting, (3) internal CDER BRC (CBER BRC) meeting, (4) internal post-BRC meeting, and (5) applicant meeting with CDER (CBER).

Another important issue aroused by the BPCI Act is the interchangeability of biosimilars. Once approved, standard generic drugs can be automatically substituted for the reference product without the intervention of the healthcare provider in many states. However, the automatic interchangeability cannot be applied to all biosimilars. In order to meet the higher standard of interchangeability, a sponsor must demonstrate that the biosimilar products can be expected to produce the same clinical result as the reference product in any given patient. 

On February 9, 2012, FDA announced the publication of three draft guidance documents to assist industry in developing follow-on biologic products, including “Scientific Considerations in Demonstrating Biosimilarity to a Reference Product”, “Quality Considerations in Demonstrating Biosimilarity to a Reference Protein Product”, “Biosimilars: Questions and Answers Regarding Implementation of the Biologics Price Competition and Innovation Act of 2009” [25,26,27]. Similar to the requirement of the WHO and EMA, a number of factors are considered important by the FDA when assessing applications for biosimilars, including the robustness of the manufacturing process, the demonstrated structural similarity, the extent to which mechanism of action was understood, the existence of valid, mechanistically related pharmacodynamics assays, comparative pharmacokinetics and immunogenicity, and the amount of clinical data and experience available with the original products. FDA is now seeking public comments on the guidance within 60 days of the notice of publication in the Federal Register. Even though the guidance does not provide clear standards for assessing biosimilar products, they are the first step towards removing the uncertainties surrounding the biosimilar approval pathway in the United States. 

#### 3.3.2. Canada (Health Canada)

Health Canada, the federal regulatory authority that evaluates the safety, efficacy, and quality of drugs available in Canada also recognizes that with the expiration of patents for biologic drugs, manufacturers may be interested in pursuing subsequent entry versions of these biologic drugs, which are called Subsequent Entry Biologics (SEB) in Canada. In 2010, Health Canada issued the “Guidance for Sponsors: Information and Submission Requirements for Subsequent Entry Biologics (SEBs)”, whose objective was to provide guidance on how to satisfy the data and regulatory requirements under the Food and Drugs Act and Regulations for the authorization of subsequent entry biologics (SEBs) in Canada [28]. 

The concept of an SEB applies to all biologic drug products, however there are additional criteria to determine whether the product will be eligible to be authorized as SEBs: (1) a suitable reference biologic drug exists that was originally authorized based on a complete data package, and has significant safety and efficacy data accumulated; (2) the product can be well characterized by state-of-the-art analytical methods; (3) the SEB can be judged similar to the reference biologic drug by meeting an appropriate set of pre-determined criteria. With regard to the similarity of products, Health Canada requires the manufacturer to evaluate the following factors: (1) relevant data for physicochemical and biological characterization; (2) analysis of the relevant samples from the appropriate stages of the manufacturing process; (3) stability data and impurities data; (4) data obtained from multiple batches of the SEB and reference to understand the ranges in variability; (5) non-clinical and clinical data and safety studies. In addition, Health Canada also has stringent post-market requirements including the adverse drug reaction report, periodic safety update reports, any suspension or revocation of NOC (notice of compliance). 

The guidance of Canada shares similar concepts and principles as indicated in the WHO’s guidelines, since it is clearly mentioned in the guidance that Health Canada has the intention to harmonize as much as possible with other competent regulators and international organizations. 

### 3.4. Asian Pacific Region (Japan & Korea)

#### 3.4.1. Japan (MHLW)

Japanese Ministry of Health, Labor and Welfare (MHLW) has also been confronted with the new challenge of regulating biosimilar/follow-on biologic products. Based on the similarity concept outlined by the EMA, Japan has published a guideline for quality, safety and efficacy of biosimilar products in 2009 [29]. The scope of the guideline includes recombinant plasma proteins, recombinant vaccines, PEGylated recombinant proteins, and non-recombinant proteins that are highly purified and characterized. Unlike in the EU, polyglycans such as low-molecular weight heparin have been excluded from the guideline. Another class of products excluded is synthetic peptides, since the desired synthetic peptides can be easily defined by structural analyses and can be defined as generic drugs. Same as the requirements by EU, the original biologic should be already approved in Japan. However, there are some differences in the requirements of stability test and toxicology studies for impurities in biosimilars between EU and Japan. A comparison of the stability of a biosimilar with the reference innovator products as a strategy for development of biosimilar is not always necessary in Japan. In addition, it is not required to evaluate the safety of impurities in the biosimilar product through non-clinical studies without comparison to the original product. According to this guideline, two follow-on biologics, “Somatropin” and “Epoetin alfa BS” have been recently approved in Japan. 

#### 3.4.2. Korea (KFDA)

In Korea, “Pharmaceutical Affairs Act” is the high level regulation to license all medicines including biologic products. The Korea Food and Drug Administration (KFDA) notifications serve as a lower level regulation. Biological products and biosimilars are subject to the “Notification of the regulation on review and authorization of biological products”. The KFDA takes an active participation in promoting a public dialog on the biosimilar issues. In 2008 and 2009, the KFDA held two public meetings and co-sponsored a workshop to gather input on scientific and technical issues. The regulatory framework of biosimilar products in Korea is a three-tiered system: (1) Pharmaceutical Affairs Act; (2) Notification of the regulation on review and authorization of biological products; (3) Guideline on evaluation of biosimilar products [30]. As Korean guideline for biosimilar products was developed along with that of the WHO’s [28], most of the requirements are similar except for that of the clinical evaluation to demonstrate similarity. The KFDA requires that equivalent rather than non-inferior efficacy should be shown in order to open the possibility of extrapolation of efficacy data to other indications of the reference product. Equivalence margins need to be pre-defined and justified, and should be established within the range which is judged not to be clinically different from reference products in clinical regards. 

## 4. Global Harmonization

According to the regulatory requirements of different regions described in the previous section, there seems to be no significant difference in the general concept and basic principles in these guidelines. There are five well recognized principles with regard to the assessment of biosimilar products: (1) the generic approach is not appropriate for biosimilars; (2) biosimilar products should be similar to the reference in terms of quality, safety, efficacy; (3) a step-wise comparability approach is required that indicates the similarity of the SBP to RBP in terms of quality is a prerequisite for the reduction of non-clinical and clinical data submitted; (4) the assessment of a biosimilar is based on a case-by-case approach for different classes of products; (5) the importance of pharmacovigilance is stressed.

However, differences have been noted in the scope of the guidelines, the choice of the reference product, and the data required for product approval. The concept of a “similar biological medicinal product” in the EU is applicable to a broad spectrum of products raging from biotechnology-derived therapeutic proteins to vaccines, blood-derived products, monoclonal antibodies, gen and cell-therapy, etc. However, the scopes of other organization or countries are limited to recombinant protein drug products. Concerning the choice of the reference product, EU and Japan require that the reference product should be previously licensed in their own jurisdiction, while other countries do not have this requirement. A detailed comparison of the guidelines of the WHO, EU, Canada, Korea and Japan for the biosimilar products is summarized in Table 2. 

In order to facilitate the global harmonization of evaluation of the follow-on biologic, the first workshop on implementing "WHO Guidelines on Evaluating Similar Biotherapeutic Products" into the regulatory and manufacturers practice at the global level was held on 24–26 August 2010 in Seoul, Republic of Korea. The workshop featured speakers from regulatory agencies from various countries, clinical and scientific experts, representatives from the biopharmaceutical industry and WHO. 

It has been recognized in the workshop that while some progress towards implementation and development of guidance documents in various countries had been made. For instance, the biosimilar guidance of Singapore and Malaysia are amended mainly based on EU’s biosimilar guidelines, while Brazil and Cuban choose the WHO and Canadian guidelines as the basis for developing regulations. However, there are also many challenges, which need to be addressed for global harmonization of the regulatory framework for licensure of biotherapeutics. For example, the manufacturing of SBPs in the Arab region is not well-controlled due to the lack of expertise in the assessment of biotechnology products and inexperience with regulatory processes. Besides, large emerging economies such as China and India are currently lagging behind in terms of their regulations and need to act rapidly in developing appropriate regulations for biosimilar product approval. 

In sum, the status of SBPs and implementation of the WHO guidelines is highly diverse worldwide, and a harmonized approach for SBPs worldwide is unlikely to occur rapidly. While some countries have developed guidelines or are developing guidelines, some countries are taking a relaxed view and are not committed on the approach to adopt for approval of SBPs. Accordingly, in order to promote the global harmonization, National Regulatory Authorities should take an active role in building capacity for regulatory evaluation of biotherapeutics; the existing guidelines should be revised as the considerable experience has been gained through Scientific Advice, Marketing Authorization Applications and Workshops; WHO should continue monitoring progress with the implementation of the guidelines on the evaluation of SBPs into regulatory and manufacturers’ practices. 

**Table 2:** Comparison of requirements for the evaluation of SBPs between different regions.

	WHO	Canada	Korea	EU	Japan
**Term**	SBPs	SEBs	Biosimilars	Biosimilars	Follow-on Biologics
**Scope**	Recombinant protein drugs			Mainly recombinant protein drugs	Recombinant protein drugs
**Efficacy**	Double blind or observer-blind； Equivalence or non-inferiority design		Equivalence design	Comparability margins should be pre-specified and justified	
**Reference Product**	Authorized in a jurisdiction with well-established regulatory framework			Authorized in EU	Authorized in Japan
**Stability**	• Accelerated degradation studies				Not necessary
	• Studies under various stress conditions				
**Purity**	• Process-related and product-related impurities				
**Manufacture**	• Same standards required by the NRA for originator products				
	• Full chemistry and manufacture data package				
**Physico-chemical**	• Primary and higher-order structure				
	• Post-translational modifications				
**Biological Activity**	• Qualitative measure of the function				
	• Quantitative measure (e.g., enzyme assays or binding assays)				
**Non-clinical studies**	• In vitro (e.g., receptor-binding, cell-based assays)				
	• In vivo (pharmacodynamic activity, at least one repeat dose toxicity study, antibody measurements, local tolerance)				
**PK study design and criteria**	• Single dose, steady-state studies, or repeated determination of PK				
	• Cross-over or parallel				
	• Include absorption and elimination characteristics				
	• Traditional 80-125% equivalence range is used				
**PD**	• Pharmacodynamic markers should be selected and comparative PK/PD studies may be appropriate				
**Safety**	• Pre-licensing safety data and risk management plan				
**Principles**	• Generic approach is not appropriate for follow-on biologic				
	• Follow-on biologic should be similar to the reference in terms of quality, safety, efficacy				
	• Step-wise comparability approach: similarity of the SBP to RBP in terms of quality is a prerequisite for reduction of non-clinical and clinical data required for approval.				
	• Case by case approach for different classes of products				
	• Pharmacovigilance is stressed				

## 5. Concluding Remarks

For the assessment of bioequivalence of generic drug products, with identified active ingredient(s) to the innovative drug products, regulatory approval pathway through the conduct of bioequivalence studies is possible only under the Fundamental Bioequivalence Assumption. For the assessment of biosimilar products, a similar Fundamental Biosimilarity Assumption could be established. It should be noted that biosimilar products are not identical but similar to the innovative products. 

As described in Section 3, most regulatory requirements for the approval of biosimilar products are similar but slightly different in the definitions of biosimilarity, the scope of the guidelines, the choice of the reference product, and the data required for product approval. Few or no discussions regarding the criteria and/or the degree of biosimilarity and/or interchangeability are given. In practice, it is a common regarding “how similar is considered highly similar?”, and sponsors ask with particular interest: “how many studies are required for the regulatory approval of biosimilar products?”. Besides, the degree of similarity may have an impact on the interchangeability of biosimilar products. As indicated in the BPCI Act, biosimilar products are considered interchangeable provided that they can produce the same therapeutic effect in any given patient. This, however, is not possible to achieve. Alternatively, we would suggest that biosimilar products could be considered interchangeable provided that they can produce the same therapeutic effect in any given patient with certain statistical assurance. 

In summary, there are still many unsolved scientific issues regarding criteria, design, and analysis for the assessment of biosimilarity and/or interchangeability of follow-on biologics. Detailed regulatory guidance for global harmonization is needed whenever possible. 
